# CYLD Inhibits the Development of Skin Squamous Cell Tumors in Immunocompetent Mice

**DOI:** 10.3390/ijms22136736

**Published:** 2021-06-23

**Authors:** Josefa P. Alameda, Verónica A. García-García, Silvia López, Ana Hernando, Angustias Page, Manuel Navarro, Rodolfo Moreno-Maldonado, Jesús M. Paramio, Ángel Ramírez, Rosa A. García-Fernández, María Llanos Casanova

**Affiliations:** 1 Molecular and Translational Oncology Unit, Centro de Investigaciones Energéticas, Medioambientales y Tecnológicas (CIEMAT), 28040 Madrid, Spain; pilar.alameda@ciemat.es (J.P.A.); veronicaArantxa@externos.ciemat.es (V.A.G.-G.); anahernandoariza@gmail.com (A.H.); a.page@ciemat.es (A.P.); manuel.navarro@ciemat.es (M.N.); rodolfo.moreno@bio-innova.es (R.M.-M.); jesusm.paramio@ciemat.es (J.M.P.); a.ramirez@ciemat.es (Á.R.); 2 Biomedical Research Institute I+12, 12 de Octubre University Hospital, 28041 Madrid, Spain; 3 Centro de Investigación Biomédica en Red de Cáncer (CIBERONC), 28029 Madrid, Spain; 4 Department of Animal Medicine and Surgery, Facultad de Veterinaria, UCM, 28040 Madrid, Spain; silvialopezcalvo@gmail.com (S.L.); ragarcia@ucm.es (R.A.G.-F.); 5 Bionomous Sàrl, PFL Innovation Park, Bâtiment, FCH-1015 Lausanne, Switzerland; 6 Bio-innova Consulting, 28049 Madrid, Spain

**Keywords:** CYLD, NF-κB, epidermal differentiation, non-melanoma skin cancer, skin tumor suppressor, apoptosis, angiogenesis, inflammation

## Abstract

Cylindromatosis (CYLD) is a deubiquitinase (DUB) enzyme that was initially characterized as a tumor suppressor of adnexal skin tumors in patients with CYLD syndrome. Later, it was also shown that the expression of functionally inactive mutated forms of CYLD promoted tumor development and progression of non-melanoma skin cancer (NMSC). However, the ability of wild-type CYLD to inhibit skin tumorigenesis in vivo in immunocompetent mice has not been proved. Herein, we generated transgenic mice that express the wild type form of CYLD under the control of the keratin 5 (K5) promoter (K5-CYLDwt mice) and analyzed the skin properties of these transgenic mice by WB and immunohistochemistry, studied the survival and proliferating characteristics of primary keratinocytes, and performed chemical skin carcinogenesis experiments. As a result, we found a reduced activation of the nuclear factor kappa B (NF-κB) pathway in the skin of K5-CYLDwt mice in response to tumor necrosis factor-α (TNF-α); accordingly, when subjected to insults, K5-CYLDwt keratinocytes are prone to apoptosis and are protected from excessive hyperproliferation. Skin carcinogenesis assays showed inhibition of tumor development in K5-CYLDwt mice. As a mechanism of this tumor suppressor activity, we found that a moderate increase in CYLD expression levels reduced NF-κB activation, which favored the differentiation of tumor epidermal cells and inhibited its proliferation; moreover, it decreased tumor angiogenesis and inflammation. Altogether, our results suggest that increased levels of CYLD may be useful for anti-skin cancer therapy.

## 1. Introduction

*CYLD* is a tumor-suppressor gene that encodes a K63 DUB enzyme. It directly regulates multiple key signaling cascades, such as the NF-κB, JNK, and the MAPK pathways, and it is a crucial regulator of diverse cellular processes such as immune responses, inflammation, death, and proliferation [1]. It was shown that genetic alterations of CYLD—rendering a catalytically inactive protein—led to the formation of tumors in patients with CYLD cutaneous syndrome [2,3,4]. Later, genome sequencing approaches have revealed somatic *CYLD* alterations in other numerous human cancers, including non-small cell lung cancer, melanoma, glioblastoma, breast, ovarian, bladder, colon, and head and neck cancer [5]. In addition, the correlation of CYLD downregulation with tumor development and progression of many types of cancers such as hepatocarcinoma, colon, and breast tumors [6,7,8] has also been described.

Among the diverse human neoplasias, NMSC are the most common malignancies. The skin consists of three layers: epidermis, dermis, and hypodermis, and it contains specialized structures, such as hair follicles (HF) and sebaceous and sweat glands. The epidermis is a stratified epithelium composed mainly of keratinocytes; among them, those of the basal layer are proliferative, move to the suprabasal layers, and gradually differentiate, giving rise to the outermost squamous cornified cell layer that is shed [9]. A balance between keratinocyte proliferation and differentiation is mandatory to maintain epidermal homeostasis; otherwise, numerous pathologies arise, including the development of NMSC. Although dysregulation of the *CYLD* gene was established to be responsible for the development of skin appendage-derived tumors in CYLD syndrome patients [2,3,4], posteriorly, our group and others reported that the catalytic inactivation of CYLD is involved in the development and progression of skin squamous cell carcinomas (SCCs) [10,11,12,13].

Basal cell carcinomas (BCCs) and SCCs represent the vast majority of the NMSC diagnosed [14] and 90% of skin cancers in the Caucasian population; of these, SCC can be very aggressive and almost 5% metastasize; therefore, due to its high incidence, the mortality caused by aggressive skin SCCs is reaching relevant numbers [15]. This and the fact that the incidence of NMSC has been increasing at an alarming rate in recent years, even in the population under 40 years of age, make NMSC an important health problem [16].

NF-κB is a ubiquitous and evolutionarily conserved transcription factor that regulates the expression of genes involved in the transformation, survival, proliferation, invasion, angiogenesis, and metastasis of tumor cells. It is composed of dimers of five members, of which p65/p50 is the predominant dimer in the skin [17]. In resting cells, NF-κB is maintained inactive, and its activation by proinflammatory signals results in the degradation of the inhibitor of NF-κB, mainly IκBα, allowing a rapid nuclear entry of the NF-κB dimers and the activation of its target genes. NF-κB has been found to be constitutively active in the tissues of most cancer patients, including those with breast, colon, stomach, oral cavity, ovary, melanoma, and lymphatic cancer [18,19,20,21,22,23]. The role for the NF-κB signaling pathway in promoting skin SCCs has also been demonstrated [24,25]: it was shown that NF-κB inhibition in keratinocytes prevented tumor development by acting both during the initiation and promotion phases of skin carcinogenesis; i.e., mice with keratinocyte-restricted p65 deficiency were resistant to 7,12-dimethylbenzanthracene/12-O-tetradecanoylphorbol-13-acetate (DMBA/TPA)-induced skin carcinogenesis, supporting the tumor-promoting role of epidermal p65 through NF-κB activation [26].

Together with the NF-κB activation, both tumor inflammation and angiogenesis are major mediators of the development and progression of skin SCCs [27,28,29]. In this context, it is relevant to mention that our previous results using xenograft carcinogenesis approaches in nude mice support the role of CYLD as a negative regulator of tumor angiogenesis in skin SCCs [10,30]; it has also been shown that CYLD acts as a negative regulator of the inflammatory response especially in the skin [31]. According to this statement, our previous studies showed that transgenic K5-CYLD^C/S^ mice lacking a functional CYLD in keratinocytes, and showing constitutive activation of NF-κB, developed chronic inflammation in the skin and spontaneous cutaneous carcinomas [11]. Other mechanisms through which our group has found that CYLD prevents the development and progression of skin SCCs are by inhibiting the proliferation and survival of epidermal tumor cells and favoring a more differentiated phenotype of cutaneous SCCs [10,11].

It is relevant that the in vivo role of CYLD as a tumor suppressor of NMSC has only been analyzed through the study of transgenic mice that either contain a functionally inactive CYLD (by expression of mutated forms of CYLD that act as dominant negative) [11,12] or that are deficient in *CYLD* [32]. However, the in vivo role of wild type CYLD in the development and progression of NMSC has not yet been fully characterized. Herein, our results obtained using immunocompetent transgenic mice show that a moderate increase in the wild type CYLD expression levels prevents the development and progression of skin squamous cell tumors, mainly through the attenuation of NF-κB activation, reduction of tumor proliferation, increased tumor cell differentiation, and inhibition of both tumor inflammation and angiogenesis. These results have important clinical significance and make *CYLD* appear as a promising therapeutic target of skin SCCs.

## 2. Results

### 2.1. Expression of the Transgene in the Skin of K5-CYLDwt Mice

**Figure 1:**
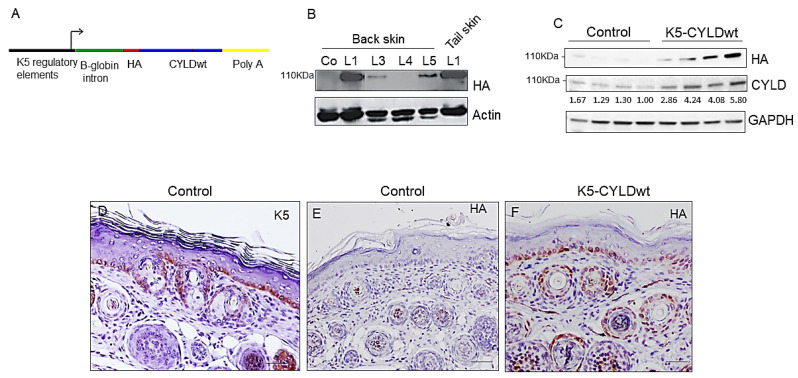
K5-CYLDwt mice express the transgenic protein following the K5 expression pattern. (**A**) Schematic representation of the K5-CYLDwt construct. The mouse complementary DNA (cDNA) tagged in 5′ with an HA epitope is expressed under the regulatory elements of the bovine K5. (**B**) Representative WB showing the transgene expression in the skin of transgenic mice of different lines (L1, L3, L4, and L5). Note that HA is not expressed in the skin of Control mice. (**C**) WB showing CYLD expression levels in the back skin of Control and K5-CYLDwt mice. Hybridization of skin of 6-month-old Control and transgenic mice of line 1 is shown. (**D**–**F**) Immunohistochemistry staining of tail skin sections from 7-day-old Control (**D**,**E**) and transgenic (**F**) mice showing the expression of K5 in the cytoplasm of keratinocytes of the basal layer and the ORS of hair follicles (**D**) and the HA expression in skin of K5-CYLDwt mice following the expression pattern of K5 (**F**). HA is not expressed in the skin of Control mice (**E**). Scale bars: 30 µm (**D**–**F**).

We have generated transgenic mice expressing the exogenous wild-type mouse CYLD protein [30] under the control of the K5 promoter. The K5-derived sequences included in this construct drive transgene expression to the skin, mainly to keratinocytes of the basal layer of the epidermis and the outer root sheath (ORS) of hair follicles [33] (Figure 1A). Five independent lines of transgenic mice were obtained. We checked by immunoblotting the expression of the transgene in the different cell lines using a specific antibody against the hemagglutinin A (HA) tag contained in the construct (Figure 1A, B); additionally, we compared CYLD expression levels in the back skin of Control and transgenic mice and found that K5-CYLDwt animals presented only a modest increase in CYLD levels; i.e., they showed a mean of 2.2-fold increase (Figure 1C). We also analyzed in situ, by immunohistochemical staining, the expression of the transgenic protein and found that it was expressed following the K5 expression pattern in both tail (Figure 1D–F) and back skin. The different lines of transgenic mice were viable, and no relevant histological differences were detected in the skin of any of them with respect to the skin histology of Control mice (Figure S1); therefore, we generically refer to them as K5-CYLDwt mice (most of the experiments were performed in line 1).

### 2.2. CYLDwt Transgene Expression Favors Epidermal Differentiation in K5-CYLDwt Mice

**Figure 2:**
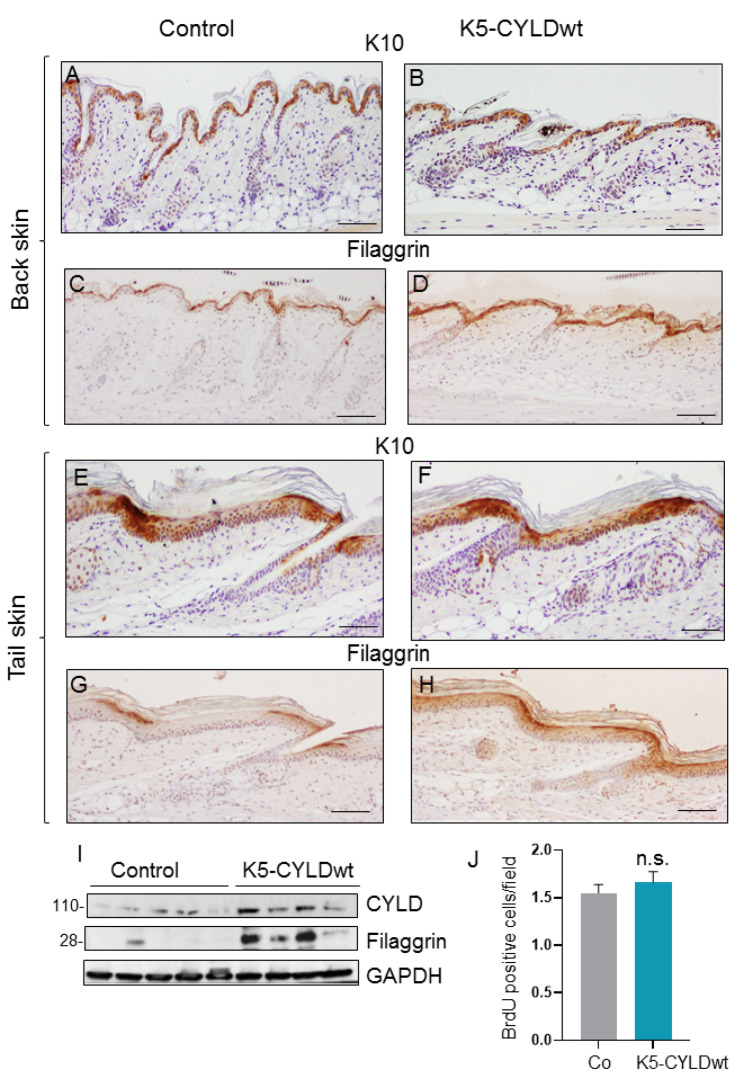
Analysis of skin differentiation and proliferation of transgenic mice. (**A**) Representative images showing the immunohistochemical analysis of the early (**A**,**B**,**E**,**F**) and terminal (**C**,**D**,**G**,**H**) epidermal differentiation in Control (**A**,**C**,**E**,**G**) and transgenic (**B,D,F,H**) mice, with specific antibodies against K10 and filaggrin, respectively. Staining of back (**A**–**D**) and tail (**E**–**H**) skin sections of 21-day-old mice is shown. Note the increased expression of filaggrin in both back and tail skin of K5-CYLDwt mice compared to Control mice (**I**). WB of total protein extracts from the back skin of Control and transgenic mice showing increased filaggrin expression in K5-CYLDwt mice. (**J**) The proliferation rate of keratinocytes was analyzed by BrdU incorporation, and no significant differences were found between the number of proliferating cells in the back skin of 6-month-old Control and transgenic mice. Five animals of each phenotype were analyzed. Data are shown as mean ± standard error of the mean (SEM) by two-tailed Mann–Whitney-test; n.s.: statistically not significant. Scale bars: 100 µm (**A**–**D**); 70 µm (**E**–**H**).

We analyzed the differentiation and proliferation properties of the skin of K5-CYLDwt mice. Epidermal differentiation of the back and tail skin was analyzed by immunohistochemical staining for early (keratin K10) or terminal (filaggrin) differentiation markers (Figure 2A–H). We observed increased filaggrin staining in both the back and tail skin of transgenic mice. However, this increase in filaggrin expression did not result in histological changes (as commented above). To further verify the increased filaggrin expression in K5-CYLDwt mice, we analyzed by Western blot (WB) the levels of filaggrin in the skin of the mice and observed augmented levels of expression in transgenic mice (Figure 2I), confirming the immunohistochemistry results. The analysis of the proliferation rate was studied by 5-bromo-2-desoxiuridina (BrdU) incorporation, and no significant differences were noticed between Control and transgenic mice (Figure 2J).

Therefore, our present results confirm, in vivo, our previous observations in human HaCat keratinocytes showing that CYLDwt overexpression favored epidermal differentiation [30].

### 2.3. The Skin of K5-CYLDwt Mice Shows Decreased NF-kB Activation and Increased Protection Against Harmful External Stimuli

**Figure 3:**
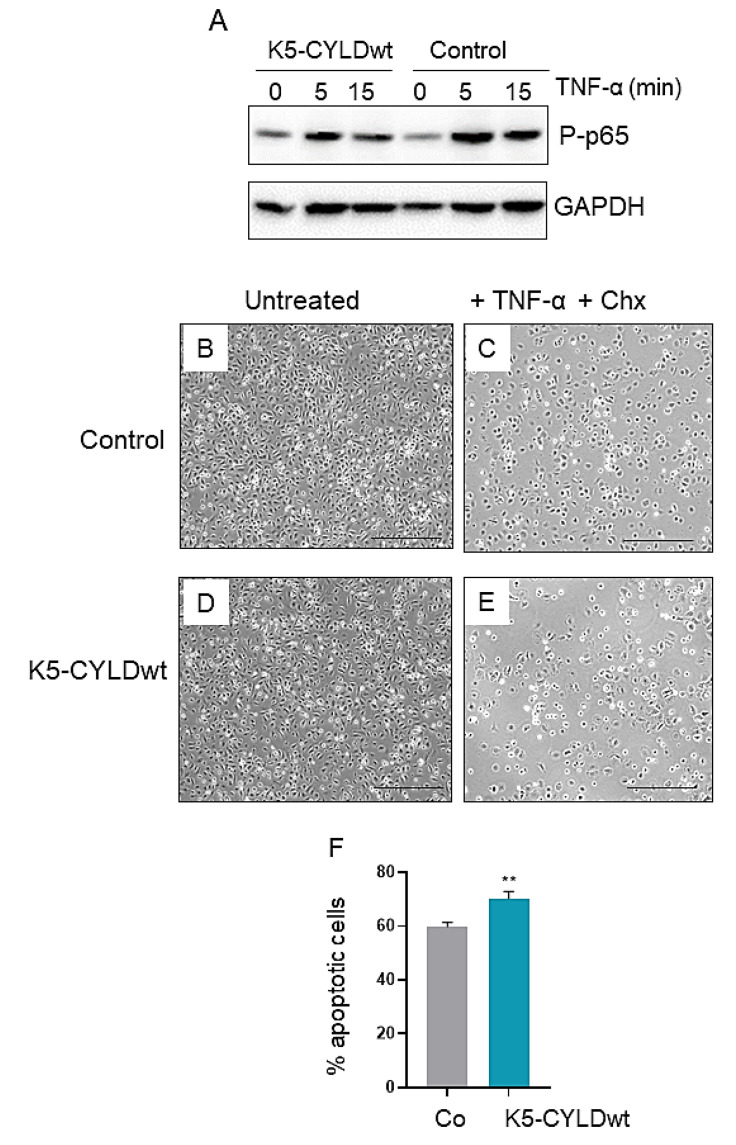
Reduced NF-κB activation of CYLDwt keratinocytes and decreased survival of transgenic keratinocytes upon exposure to an apoptotic stimulus. (**A**) Kinetics of p65 phosphorylation in the back skin of 3-day-old Control and transgenic mice inoculated with TNF-α for the indicated times. Control (**B**,**C**) and transgenic (**D**,**E**) keratinocytes were grown in the absence (**B**,**D**) or presence (**C**,**E**) of TNF-α and cycloheximide (Chx) for 15 h. Representative images showing increased number of viable cells in Control plates are shown. Scale bars (**B**,**E**): 1000 µm. (**F**) Graphical representation showing the significantly higher percentage of transgenic keratinocytes dying by apoptosis. Data are shown as mean ± SEM; ** *p* < 0.01 by two-tailed *t*-test with Welch’s correction.

CYLD is mainly known for its function as a negative regulator of NF-kB activation [2,3,4]; thus, we analyzed the kinetic of NF-kB activation (measured as levels of phosphorylated p65 (P-p65)) in the skin of transgenic mice in response to an exogenous stimulus; for this purpose, TNF-α was inoculated in the back skin of 3-day-old mice, and the phosphorylation of p65 was measured at different time points. We found that the skin of Control mice showed a greater activation of NF-kB at early time points after TNF-α treatment (5′) that lasted for longer periods of time (15′) (Figure 3A); in contrast, the activation of NF-kB exhibited by transgenic mice was lower at early time points (5′) and almost imperceptible at longer times (15′).

**Figure 4:**
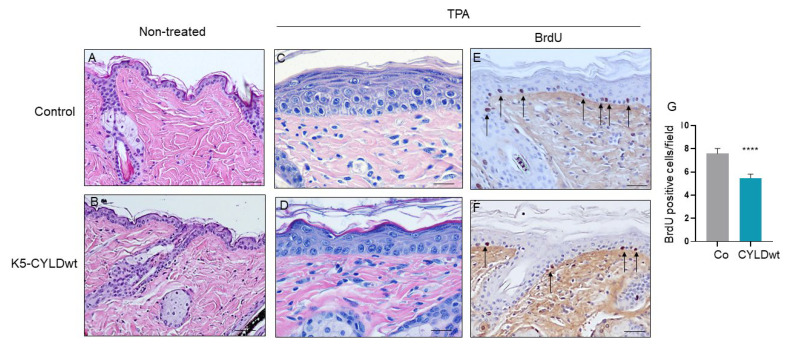
Diminished epidermal hyperplasia in the skin of K5-CYLDwt mice subjected to TPA treatment. (**A**–**D**) Representative back skin sections from Control (**A**,**C**) and K5-CYLDwt (**B**,**D**) mice stained with hematoxylin and eosin (H&E). (**A**,**B**) Untreated skins. The vehicle (acetone) was applied for 3 weeks in the back skin of mice, and no relevant differences were found between the histology of the back skin of Control (**A**) and transgenic (**B**) mice. (**C**,**D**) Hyperplastic epidermis induced by topical application of TPA. Observe the modest increase in the number of epidermal layers that TPA elicits in the skin of transgenic mice (**D**). (**E**,**F**) Representative images showing BrdU incorporation in the skins of Control (**E**) and K5-CYLDwt (**F**) mice treated with TPA. Note the lower number of BrdU-positive cells per field detected in the epidermis of transgenic mice. (**G**) Graphical representation of the number of BrdU-positive cells per field in the back skin of mice of both genotypes. Data are shown as mean ± SEM **** *p* < 0.0001 by two-tailed Mann–Whitney test. Scale bars: 50 µm (**A**,**B**); 20 µm (**C**–**D**); 30 µm (**E**–**F**).

Since we found lower NF-κB activation in the skin of K5-CYLDwt mice, we wondered what would be the response of keratinocytes to different insults. Among the most relevant functions of NF-κB activation are the induction of survival proteins and resistance to apoptosis as well as promotion of cell proliferation. Thus, we tested the response of both Control and transgenic keratinocytes to two different types of exogenous insults: first, we studied the response of primary keratinocytes from newborn mice of both genotypes to apoptosis induced by growth in the presence of TNF-α and Chx [34] (Figure 3B–E), and we observed that K5-CYLDwt keratinocytes exhibited increased susceptibility to apoptosis, showing a significant reduction in the number of viable cells (Figure 3F). Next, we applied multiple doses of the mitogen agent TPA to the back skin of 6-month-old Control and transgenic mice. Both types of mice showed in the absence of TPA treatment an epidermis formed by two to three layers of keratinocytes, and TPA applications led to epidermal hyperplasia in mice of both genotypes; however, compared with the striking epidermal hyperplasia that the TPA treatment provoked in Control mice, consisting of seven to ten layers of keratinocytes (Figure 4A,C), the skin of K5-CYLDwt mice exhibited lower epidermal hyperplasia, consisting of just five to seven layers of keratinocytes (Figure 4B,D). These results are consistent with the reduced number of BrdU-positive cells detected by immunohistochemistry in the skin of TPA-treated transgenic mice (Figure 4E–G). Therefore, our results suggest that a modest increase in the levels of expression of CYLD might prevent the excessive hyperproliferation of keratinocytes and make them more prone to death by apoptosis when subjected to aggressions.

### 2.4. Inhibition of Skin Squamous Cell Tumor Development in K5-CYLDwt Mice

**Figure 5:**
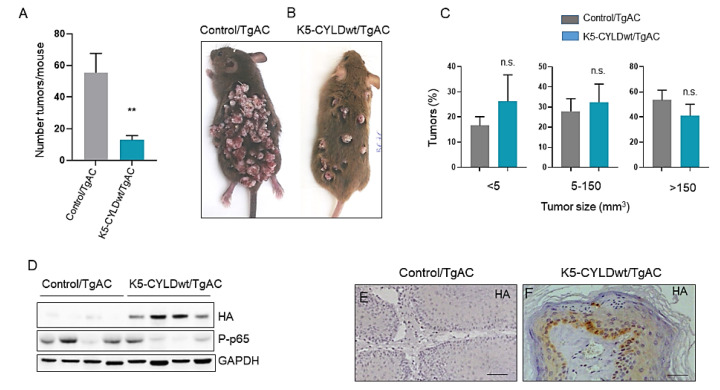
Inhibition of skin tumor development in K5-CYLDwt mice. (**A**) Graphical representation of the tumor multiplicity in Control/TgAC and K5-CYLDwt/TgAC mice showing the decrease in the number of tumors developed in the latter. Data are shown as mean ± SEM; ** *p* < 0.01 by two-tailed unpaired *t* test with Welch’s correction; n = 10 mice/genotype. (**B**) Representative images showing the reduction in the number of tumors in the skin of K5-CYLDwt/TgAC mice. (**C**) Representation of the percentage of tumors of the indicated size in each group of mice. Note a tendency to reduced size in K5-CYLDwt/TgAC tumors. Data are shown as mean ± SEM by two-tailed Mann–Whitney test; n.s.: statistically not significant. (**D**) WB showing that tumors arisen in K5-CYLDwt/TgAC mice expressed the transgene and showed inhibition of the activation of NF-κB. (**E**,**F**) Immunostaining with a specific anti-HA antibody showing the expression of the transgene in K5-CYLDwt/TgAC tumors (**F**). Scale bars: 30 µm (**E**,**F**).

**Figure 6:**
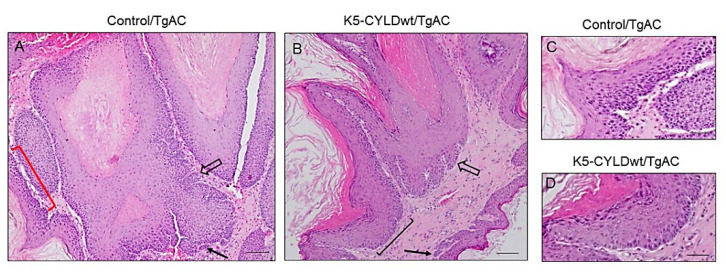
Histology of tumors developed by Control/TgAC and K5-CYLDwt/TgAC mice. Representative H&E staining showing the histology of tumors collected 17 weeks after TPA treatment. (**A**,**B**) Both tumors have a papillary exophytic growth with high cellularity and hyperkeratosis. Back arrows point to microinfiltration foci; open arrows indicate epithelial extensions that project into the dermis (rete pegs); red bracket in (**A**): basal hyperplasia; black bracket in (**B**): non-hyperplastic basal layer. (**C**,**D**) Magnification showing the basal hyperplasia in the Control/TgAC tumor (**C**) versus no hyperplasia in the basal layer of the K5-CYLDwt/TgAC tumor (**D**). Scale bars: 60 µm (**A**,**B**); (**C**,**D**) 30 µm.

The results commented above suggested that a moderate increase in CYLD expression could also provide protection to keratinocytes against carcinogenic insults; therefore, we analyzed whether wild type CYLD could prevent in vivo, in our immunocompetent transgenic mouse model, the development of skin squamous cell tumors. Chemical skin carcinogenesis was performed in both types of mice (Control and K5-CYLDwt mice) that had been previously bred with TgAC animals, which carry an activated Ha-*ras* transgene that triggers the classic skin tumor initiation event [35,36]. Double transgenic (K5-CYLDwt/TgAC) and Control/TgAC mice were treated with TPA, which promotes the expansion of *ras*-activated cells. In this cancer model, the resulting tumors developed in the form of papillomas that may regress or progress to SCC [37], allowing the study of early and late events in cancer development and progression. We found no significant differences in the onset of tumor development between Control/TgAC and K5-CYLDwt/TgAC mice; tumor appearance was visible 9 weeks post-TPA treatment. However, we found that tumor multiplicity (number of tumors/mice) was significantly higher in Control/TgAC mice, as can be seen in the graph (Figure 5A), as well as in the macroscopic image of these mice that shows the notable difference in the number of tumors developed in each type of mouse (Figure 5B). Additionally, we found a tendency toward the development of small-sized tumors in K5-CYLDwt/TgAC mice, with tumors larger than 150 mm^3^ being preponderant in Control/TgAC mice, whereas the numbers of small (<5 mm^3^) and medium-sized (5–150 mm^3^) tumors were higher in K5-CYLDwt/TgAC mice (Figure 5C). We verified by WB and immunostaining the expression of the transgene in the tumors of K5-CYLDwt/TgAC mice (Figure 5D,F). In addition, we detected lower levels of NF-kB activation in K5-CYLDwt/TgAC tumors (Figure 5D). Histologically, the squamous cell skin tumors developed by the two groups of mice were benign tumors; they presented an exophytic growth, with elevated cellularity and papillary projections containing a central axis with dense fibrovascular stroma and occasional epithelial invaginations into the dermis (rete pegs) and foci of microinfiltration of neoplastic epithelial cells surrounding by stroma with no basal membrane discontinuity (Figure 6). However, the presence of more frequent rete pegs and microinfiltration foci in Control/TgAC tumors than in the K5-CYLDwt/TgAC ones was relevant (Figure 6A,B); also, marked areas of basal hyperplasia were observed in tumors developed in Control/TgAC mice (Figure 6A,C), altogether suggesting their greater tumor progression.

### 2.5. Skin Squamous K5-CYLDwt/TgAC Cell Tumors Show Reduced Proliferation and Increased Differentiation

**Figure 7:**
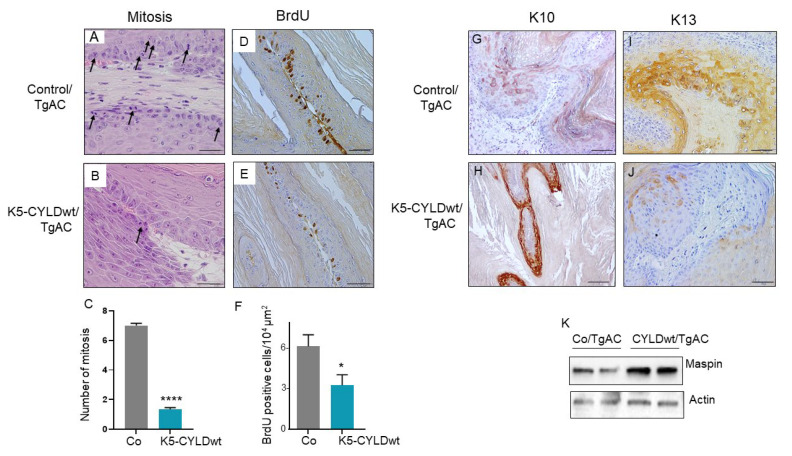
K5-CYLDwt/TgAc tumors are less proliferative and more differentiated. (**A**,**B**) Representative pictures showing increased number of mitosis in Control/TgAC tumors. (**C**) Graph showing the significant increase in the number of mitosis/µm^2^ in Control/TgAC tumors. (**D**,**E**) Immunostaining with an anti-BrdU antibody showing increased number of proliferating cells in Control/TgAC tumors (**D**) than in K5-CYLDwt/TgAC tumors (**E**). (**F**) Graph showing the diminished number of BrdU-positive cells/µm^2^ in K5-CYLDwt/TgAC tumors. (**G**,**H**) Observe the weak and discontinuous expression of K10 in Control/TgAC tumors (**G**) versus the strong and continuous expression in K5-CYLDwt/TgAC tumors (**H**). (**I**,**J**) K13 immunostaining. Note the induction of K13 in Control/TgAC tumors (**I**), while only sporadic K13 positive cells were detected in K5-CYLDwt/TgAC tumors (**J**). (**K**) WB showing increased Maspin expression in K5-CYLDwt/TgAC tumors. Data are shown as mean ± SEM; **** *p* < 0.0001; * *p* < 0.05 by two-tailed t-test with Welch’s correction. Mitosis: n = 79 tumors from five different Control/TgAC mice; n = 32 tumors from five different K5-CYLDwt/TgAC mice. BrdU staining: seven tumors from five mice of each genotype. Scale bars: 25 µm (**A**,**B**); 60 µm (**D**,**E**); 50 µm (**G**–**J**).

To determine the possible causes of the decrease in volume of tumors developed in K5-CYLDwt/TgAC mice, we studied the proliferative capacity of neoplastic cells by analyzing the mitotic and BrdU incorporation indexes. We found that the number of mitosis per field was lower in the K5-CYLDwt/TgAC tumors than in the Control/TgAC ones (Figure 7A,B), showing a statistically significant difference. In agreement with this observation, the immunostaining with an anti-BrdU antibody showed a lower number of proliferative cells in tumors developed in K5-CYLDwt/TgAC mice, which also indicated less cell proliferation in epidermal tumors expressing increased levels of CYLD wild type (Figure 7D–F). Apoptosis was almost not detected in any of the tumors from Control/TgAC and K5-CYLDwt/TgAC mice (analyzed by active Caspase 3 immunohistochemistry, data not shown). Altogether, our results suggest that the smaller size of K5-CYLDwt/TgAC tumors is due to a diminished cell proliferation.

The analysis of the expression of different keratins in carcinomas is commonly used as a marker of the degree of tumor differentiation as well as a marker of tumor progression. Thus, we performed immunostainings against keratins K10—a reliable marker of well differentiated tumors—and K13—a keratin not expressed in normal skin and considered a marker of tumor progression when it is aberrantly expressed in skin tumors [33]. Our results showed that Control/TgAC tumors expressed lower levels of K10 than K5-CYLDwt/TgAC ones, while K13 was highly expressed in tumors from Control/TgAC mice and hardly detected in K5-CYLDwt/TgAC tumors (Figure 7G–J). Therefore, the expression pattern of these keratins indicated that tumors from K5-CYLDwt/TgAC mice were more differentiated and showed fewer signs of progression, suggesting a better prognosis. We and others have recently described Maspin as an indicator of skin squamous cell tumors aggressiveness, in which lower levels of Maspin were associated with more aggressive tumors [30,38,39]; in addition, we reported that the exogenous overexpression of CYLDwt in skin SCC cell lines correlated with increased levels of Maspin [40]. We analyzed Maspin expression by WB and found increased levels in K5-CYLDwt/TgAC tumors (Figure 7K), which together with the data shown above also suggest a reduced malignancy of tumors with higher CYLD expression.

### 2.6. Skin Squamous Cell Tumors Developed in K5-CYLDwt/TgAC Mice Show Less Tumor Angiogenesis and Lower Inflammation

**Figure 8:**
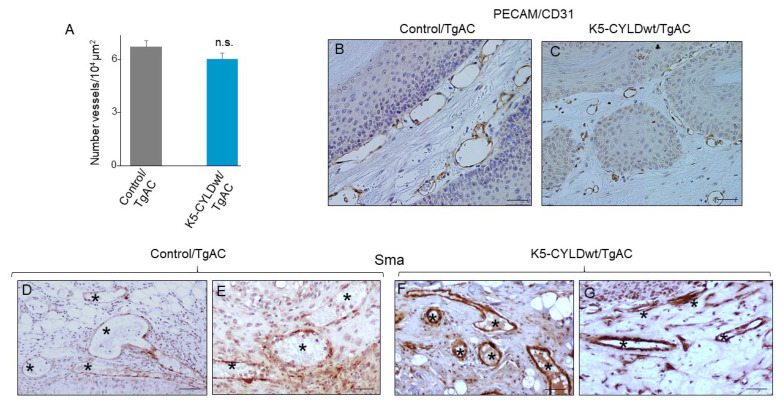
Smaller size and increased maturity of blood vessels in K5-CYLDwt/TgAC skin squamous cell tumors. (**A**) Graph showing the number of blood vessels/µm^2^ found in Control/TgAC and K5-CYLDwt/TgAC tumors; no significant differences were detected. Data are shown as mean ± SEM by two-tailed t-test; n = 6 tumors/genotype; n.s.: statistically not significant. (**B**,**C**) Representative images of the immunostaining with PECAM (CD31) antibody showing the network of blood vessels in Control/TgAC and K5-CYLDwt/TgAC tumors. Observe the presence of lacunar vessels of large diameter in Control/TgAC tumors (**B**) versus vessels of small diameter in K5-CYLDwt/TgAC tumors (**C**). **(D**–**G**) Immunohistochemistry with an anti-Sma antibody reveals a weak and discontinuous staining of blood vessels in Control/TgAC tumors (**D**,**E**), versus a strong and continuous staining in vessels of K5-CYLDwt/TgAC tumors (**F**,**G**). Scale bars: 40 µm (**B**–**C**); 20 µm (**D**–**G**). The symbol (*) indicates the light of the blood vessels in (**D**–**G**).

A relevant factor that makes tumor growth and progression possible is a permeable network of blood vessels. Analysis of the vasculature in both types of tumors showed no significant differences in blood vessel density between Control/TgAC and K5-CYLDwt/TgAC tumors (Figure 8A); however, there were important differences in the size of the vessels, being those of K5-CYLDwt/TgAC tumors of smaller diameter, as observed through the analysis of blood vessels with PECAM/CD31 antibody (compare Figure 8B,C). In addition, immunostaining with a specific antibody against α-smooth muscle actin (Sma) showed an intense and continuous expression in the blood vessels of K5-CYLDwt/TgAC tumors, suggesting a mature and impermeable vasculature phenotype, while there was weak and discontinuous staining in the vessels of Control/TgAC tumors, indicating that the blood vessels of Control/TgAC tumors were more immature (Figure 8D–G). This is consistent with the bleeding observed, i.e., erythrocyte extravasation from the blood vessels, that was more common in tumors originated in Control/TgAC animals (51% of tumors presented areas of bleeding) than in those from K5-CYLDwt/TgAC mice (only 38% of tumors showed bleeding) (data not shown).

**Figure 9:**
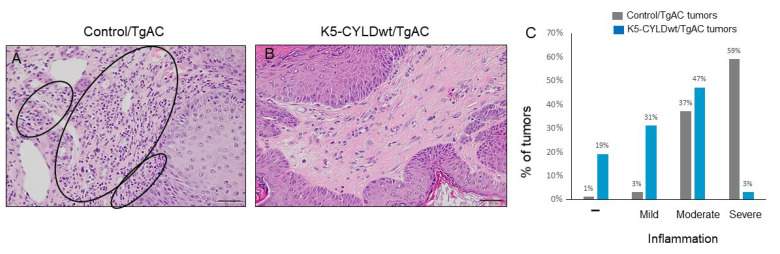
Reduced inflammation in K5-CYLDwt/TgAC tumors. (**A**) Circles indicate abundant inflammatory infiltrates in the stroma of Control/TgAC tumors in contrast to the lesser inflammation detected in K5-CYLDwt/TgAC tumors (**B**). (**C**) Graph showing that most of Control/TgAC tumors (the 96%) presented severe or moderate inflammation, while only 4% did not show important inflammation; by contrast, 50% of tumors developed in K5-CYLDwt/TgAC mice showed low or no signs of inflammation. Data are shown as mean ± SEM by two-tailed t-test; n = 79 Control/TgAC tumors and n = 32 K5-CYLDwt/TgAC tumors. Scale bars: 50 µm (**A**,**B**).

Another difference found between tumors developed in Control/TgAC and K5-CYLDwt/TgAC mice was related to the presence of inflammatory cells, mainly lymphocytes and some plasma cells. We observed that 59% of Control/TgAC tumors and only 3% of K5-CYLDwt/TgAC tumors presented severe inflammation (Figure 9A,B); conversely, while 50% of K5-CYLDwt/TgAC tumors showed no or mild inflammation, only 4% of Control/TgAC tumors lacked inflammation (Figure 9C). Therefore, these data indicate that a moderate increase in the levels of expression of CYLD significantly inhibited inflammation in skin squamous cell tumors.

## 3. Discussion

The incidence of cutaneous squamous cell carcinomas has been increasing over the past several decades, with more than 700,000 cases annually [41]. Despite surgery, radiation, and chemotherapy, SCC cells can escape treatment and even originate metastasis, increasing morbidity and mortality. To obtain a deeper understanding of possible efficient therapies for skin SCCs, it is important to find targets that could eradicate SCCs or prevent their progression. In this context, our results showing that a modest increase in the expression levels of CYLD in the skin of K5-CYLDwt mice is able to inhibit the development and progression of skin squamous cell tumors is a relevant finding for future clinical approaches.

Our previous studies of skin carcinogenesis in nude mice showed that the lack of the catalytic function of CYLD increased the aggressiveness of cutaneous SCCs [10,30]; i.e., we found that the expression of the mutant CYLD^C/S^, which acts as a dominant negative by inhibiting the deubiquitination function of the endogenous CYLD, enhanced tumor cell proliferation, survival, and tumor angiogenesis (the latter considered a prominent feature of skin tumor progression) in both mouse and human skin SCCs [10,30,42]. Additionally, we showed that the overexpression of wild type CYLD in human skin SCC cells led, in xenograft assays in nude mice, to the development of more differentiated tumors with less efficient angiogenesis [30]. However, the in vivo role of CYLD as a tumor suppressor of skin squamous cell tumors in immunocompetent mice has not yet been elucidated. Here, we show that a modest increase in CYLD expression in K5-CYLDwt mice does not have any deleterious effect on normal keratinocytes, although it confers to them an increased differentiation capacity and attenuates their responses to environmental stresses on the skin (such as TNF-α + Chx or TPA application), mainly through increased cell apoptosis and reduced proliferation. On the contrary, we show that an increase in CYLD levels exerts a relevant role counteracting the damaging effects of transformed keratinocytes, as it largely prevents the development of squamous cell epidermal tumors, and the few tumors that arise exhibit signals of better prognosis, such as smaller size, decreased proliferation, more differentiated phenotype, lower number of inflammatory cells, and a network of small and mature blood vessels.

Taken together, our results suggest that wild type CYLD is able to repress skin squamous cell tumor development and progression in vivo, in immunocompetent transgenic mice, offering a powerful anti-cancer target against this type of skin neoplasia. In this context, it will be interesting to analyze the behavior of K5-CYLDwt/TgAC tumors at longer times of evolution, since our previous studies assigned a role to CYLD as a suppressor of lung metastasis of skin SCCs [40]. In addition, our results showed the relevant role of Maspin expression in the inhibition of metastasis by CYLD.

### 3.1. Inhibition of NF-kB Activation in Keratinocytes and Skin Squamous Cell Tumors of K5-CYLDwt/TgAC Mice

Our data suggesting the role of *CYLD* as a tumor suppressor of skin squamous cell tumors in vivo are in agreement with those of *Cyld*-/- mice, in which other authors found that mice lacking the *Cyld* gene exhibited increased susceptibility to skin cancer development in chemical carcinogenesis experiments [32]; they also agree with our recent results showing that transgenic mice lacking the deubiquitinase function of CYLD in keratinocytes, K5-CYLD^C/S^ mice, spontaneously develop skin tumors [11].

We have found that attenuation in keratinocytes of the activation of NF-κB emerges as a very likely mechanism through which CYLD exerts its function as a tumor suppressor of skin squamous cell tumors. Indeed, many different human cancers, both hematological malignancies and solid tumors, have been linked to constitutive NF-kB activation, including skin SCCs [24]. In addition, it was specifically demonstrated that the inhibition of the signaling through this pathway inhibited the development of SCCs in DMBA/TPA chemical skin carcinogenesis assays; i.e., blockage of NF-kB activation in keratinocytes of transgenic mice prevented skin tumorigenesis by acting both during the initiation and promotion phases [26]. We have also observed diminished activation of NF-kB not only in keratinocytes but in K5-CYLDwt/TgAC tumors, which is very relevant, since diminished NF-κB signaling inhibits responses such as inflammation, cell proliferation, and angiogenesis, which cause tumor development and progression [43]. As a result, in agreement with all these evidences indicating the pro-tumoral role of NF-kB in cancer, we found that the rare squamous cell skin tumors developed from keratinocytes expressing slightly higher levels of wild type CYLD present a better prognosis.

### 3.2. More Differentiated and Less Proliferative Phenotype of K5-CYLDwt/TgAC Tumors

In addition to the reduced number and size of K5-CYLDwt/TgAC tumors, the keratin pattern expression showed that they were more differentiated and had almost no expression of K13, which is a keratin indicative of malignant progression of NMSC [10]. These results are consistent with the role of CYLD as a positive regulator of epidermal differentiation that we previously described in human HaCaT keratinocytes as well as in mouse and human tumor epidermal cells [30]. Additionally, K5-CYLDwt/TgAC tumors are smaller than Control/TgAC tumors, which could be due to the lower levels of proliferation detected in these tumors as well as to the minor development of a network of blood vessels that would impair the nutrition of tumor cells. The role of CYLD as an inhibitor of keratinocyte proliferation was previously reported by our group and others in xenografts assays and in *cyld*-deficient mice [10,32].

### 3.3. Diminished Tumor Inflammation and Angiogenesis in K5-CYLDwt/TgAC-Derived Tumors

A relevant feature of K5-CYLDwt/TgAC tumors is its lower inflammation and angiogenesis compared to tumors arisen in Control/TgAC mice. The activation of NF-κB is an established pathway in the mediation of inflammation, which in turn is a major mediator of tumor development and progression of mouse and human skin SCCs [27,44]. Among other effects, inflammatory cells promote angiogenesis, favoring the development of the vascular network in tumors [45]. Angiogenesis is a relevant process that plays a key role in tumor progression, since a robust network of blood vessels makes possible the supply of oxygen and nutrients needed for cancer progression [42,46]. Therefore, the lower number and better prognosis of K5-CYLDwt/TgAC tumors could be the consequence of the decreased inflammatory environment of the tumor cells as well as the reduced dimensions and mature nature of their blood vessels. In turn, the reduced size of the blood vessels could contribute to the more differentiated phenotype of K5-CYLDwt/TgAC tumors, as the enlargement of angiogenic blood vessels is the main vasculature change that allows tumor progression [42]. Our present data showing the anti-angiogenic role of CYLD in skin squamous cell tumors developed in immunocompetent mice are in agreement with those that we obtained in studies of xenograft carcinogenesis in nude mice, showing that CYLDwt overexpression in SCCs reduced the diameter of the tumor blood vessels and augmented its maturity [30]. They are also consistent with our data showing that the impaired catalytic function of CYLD in tumor epidermal cells enhanced angiogenesis in both mouse and human skin SCCs, contributing to its malignant progression and metastatic behavior [10,30,40]. Additionally, we have recently reported that the main mechanisms that led to the development of spontaneous skin tumors in K5-CYLD^C/S^ mice were the chronic activation of NF-κB and constitutive inflammation in the skin of these mice [11]. Therefore, our data in the present work suggest that a moderate increase in CYLD levels in keratinocytes is able to inhibit inflammation and angiogenesis in skin squamous cell tumors and then act as a tumor suppressor of this type of NMSC in mice with an undamaged immune system.

### 3.4. Relevance of Wild Type CYLD Expression for Clinical Management of Skin SCCs

Our results suggest that a moderate increase in CYLD levels could be useful for anti-cancer therapy. So far, evidence of the regulation of *CYLD* expression by pharmacological agents is scarce in the literature, but it has been reported that it may be possible to indirectly regulate CYLD levels by agents acting on CYLD regulatory factors, such as Serum Response Factor (SRF) or kinase inhibitors [1]; also, CYLD levels may be indirectly increased by allosteric inhibition of Caspase 8 (which cleaves CYLD) by the pan-caspase inhibitor zVAD-FMK [47]. Caspase 8 is upregulated and localized to the nucleus in multiple human cancers, correlating with resistance to therapy and poor clinical outcome: it promotes NF-κB-dependent expression of several cytokines, angiogenesis, and tumorigenesis [48], suggesting that the inhibition of Caspase-8 could be an interesting promising regulator of CYLD levels in cancer. In addition, the role of p38 as a mediator of the positive regulation of CYLD expression by SRF has been described [49], suggesting that the use of p38 inhibitors could be an appropriate approximation to favor CYLD-mediated inhibition of skin squamous cell tumor development, since the use of novel, isoform-specific p38 inhibitors has shown that the abrogation of p38δ activity in keratinocytes is accompanied by the simultaneous overactivation of p38α, which in turn can lead to increased CYLD expression through SRF [50,51,52].

It is now clear that cancerous phenotypes result from the dysregulation of more than 500 genes at multiple steps in cell signaling pathways [53,54]. This indicates that the inhibition of a single gene product or a single cell signaling pathway is unlikely to prevent or treat cancer. However, most current anti-cancer therapies are based on the modulation of a single target. Therefore, our results showing that the modulation of a single gene, *CYLD*, is able to regulate multiple targets in skin squamous cell tumors are very relevant from a clinic point of view and would provide a huge advantage as a cancer treatment, since we show how CYLD controls many of the major dysregulated features in cancer, such as cell proliferation, differentiation, cell survival, angiogenesis, and inflammation.

## 4. Materials and Methods

### 4.1. Generation of Transgenic Mice

HA-tagged murine CYLD [30] was placed under the control of a 5.2 kb-upstream fragment of bovine K5 promoter and a rabbit β-globin intron [33] (Figure 1A). Transgenic mice were generated by the microinjection of this construct into C57BL/6 Jx DBA/2J (B6D2) F2 embryos using standard techniques and transgenic lines were maintained by crossing with B6D2F1 mice. Mice were genotyped by PCR analysis of tail genomic DNA using primers specific for the rabbit β-globin intron. Non-transgenic littermates were used as control animals.

### 4.2. Histology and Immunohistochemical Staining

Skin and tumors were fixed in 10% buffered formalin and embedded in paraffin. Sections were stained with H&E, and histopathological evaluation was performed by an experimented observer veterinarian expert in animal pathology (R.A.G.F.). Immunostaining was performed using antibodies against HA (3724, Cell Signaling Technology), K5 (PRB-160P), K10 (PRB-159P), and filaggrin (PRB-417P) (Biolegend, San Diego, CA, USA); K13 (AE8; Abcam, Cambridge, UK); PECAM/CD31 SC-1506 (Santa Cruz Biotechnology, Inc., Santa Cruz, CA, USA); CYLD (SAB4200061) and sma (C-6198) (Sigma-Aldrich, St Louis, MO, USA); and BrdU (11170376001; Roche, Mannheim, Germany). Sections were incubated with a biotinylated secondary antibody and then with streptavidin conjugated to horseradish peroxidase (DAKO A/S, Glostrp, Denmark). Antibody localization was determined using 3,3-diaminobenzidine (DAB) (Vector Laboratories; Burlingame, CA, USA).

### 4.3. Western Blot Analysis

Protein extracts were obtained from pieces of back skin or tumors. Total protein extracts (40 μg) were subjected to SDS/PAGE. The separated proteins were transferred to nitrocellulose membranes (Amersham, Arlington Heights, IL, USA; BioRad, France) and probed with antibodies against CYLD (SAB4200061, Sigma-Aldrich, St Louis, MO, USA); HA (3724) and P-p65 (3033) (Cell Signaling Technology, Danvers, MA, USA); maspin (PA5-35104; Invitrogen, Thermo Fisher Scientific, Waltham, MA, USA); actin (sc-1616); GAPDH (sc-25778) (Santa Cruz Biotechnology, Inc., Santa Cruz, CA, USA), and filaggrin (Biolegend, San Diego, CA, USA). In all cases, samples were subjected to luminography with the Supersignal West Pico Chemiluminescent Substrate (Pierce Biotechnology, Inc., Rockford, IL, USA).

### 4.4. TNF-α In Vivo Treatment

Three-day-old mice were subcutaneously injected with 20 mg/Kg of human TNF-α (Sigma-Aldrich, St Louis, MO, USA) in PBS or with PBS (control). After the indicated times, mice were sacrificed, skin samples removed, and proteins extracted. Experiments were carried out on two different litters of mice, each composed of control and transgenic littermates.

### 4.5. TPA Hyperplastic Treatment

Control and K5-CYLDwt 5-month-old mice (10 mice of each genotype) were used. Shaved dorsal skins of five mice of each genotype were treated twice a week with 5 µg of TPA (Sigma-Aldrich, St Louis, MO, USA)/200 µL acetone for 3 weeks. Five mice of each genotype were treated in a similar way just with the vehicle (acetone). Mice were sacrificed 24 h after the last application.

### 4.6. Primary Keratinocyte Cultures

For primary mouse keratinocyte cultures, the skin epidermis of P1 or P2 control and transgenic littermates was separated from the dermis by trypsinization with 0.25% trypsin (Gibco, Thermo Fisher Scientific, Waltham, MA, USA) in phosphate-buffered saline. Primary keratinocytes were seeded at a density of 3 × 10^6^ cells per 60 mm diameter Petri dish in Ca^2+^ -free and Mg^2+^ -free Eagle’s minimal essential medium (BioWhittaker, Walkersville, MD, USA; Cambrex, Charles City, IA, USA) supplemented with 4% Chelex-treated (Bio-Rad, Hercules, CA, USA) fetal bovine serum (Cultek, Madrid, Spain) and 0.2 mM Ca^2+^. After 16 h, the medium was replaced by fresh medium containing 0.05 mM Ca^2+^ and 10 ng/mL epidermal growth factor (Sigma-Aldrich, St Louis, MO, USA).

### 4.7. Apoptosis Assays in Primary Keratinocytes

Apoptosis assays were performed as described [34]. Briefly, subconfluent 60 mm dishes of primary keratinocytes were treated for 15 h with 10 ng/mL TNF-α (Sigma-Aldrich, St Louis, MO, USA) and 10 µg/mL cycloheximide (Sigma-Aldrich, St Louis, MO, USA). Cells were trypsinized, and the percentages of viable cells were obtained by counting the cells in a Neubauer chamber. Experiments were performed 3 times per duplicate; in either experiment, pools from 3 to 6 animals of each phenotype were cultured together.

### 4.8. Carcinogenesis Assays

Carcinogenesis experiments in Tg.AC background were performed. Briefly, female homozygous v-Ha-ras transgenic Tg.AC mice [29,30] were mated with K5-CYLDwt males. Double transgenic K5-CYLDwt/TgAC and Control/TgAC 9-week-old mice (10 animals per group) were shaved and topically treated twice weekly with 5 μg of TPA (Sigma) in 200 μL of acetone for 9 weeks according to standard protocols [35]. Two different carcinogenesis assays were performed with a total of 10 animals of each genotype treated (5 animals of each phenotype in each carcinogenesis assay). Tumors were measured with an external caliper, and volume was calculated as (4/3) π (width/2)^2^ (length/2). Tumors were harvested 17 weeks after TPA treatment. For calculation of the number of mitosis, histological sections from 6 to 8 different tumors of each genotype were valuated. Ten non-consecutive fields were counted at 40× magnification by two different blinded researches. Inflammation was assessed according to the percentage of the stromal area that showed the presence of inflammatory cells: mild, 1–10%; moderate, 11–49%; and severe, ≥50%. The number of blood vessels was calculated with the CellSens program of images analysis (Olympus America Inc., Center Valley, PA, USA).

### 4.9. BrdU Labeling

Mice received an intraperitoneal injection of BrdU 120 mg/kg body weight 1 h before sample harvesting. Histological sections of the back skin of 5–6 mice of each genotype were evaluated: the presence of BrdU-positive cells in 25–35 fields were counted at 10× magnification by two different researches. In the case of tumors, histological sections of 8–10 tumors of each genotype were also evaluated; in those sections, 10 fields at 40× magnification in each tumor were analyzed by counting the number of BrdU-positive cells.

## Data Availability

This study do not report any data. No datasets were generated or analyzed during the current study.

## Supplementary Materials

The following are available online at https://www.mdpi.com/article/10.3390/ijms22136736/s1, Figure S1: No histological changes are observed in the skin of K5-CYLDwt mice.

## Abbreviations

BCC	basal cell carcinomas
BrdU	5-bromo-2-desoxiuridina
Chx	cycloheximide
DMBA	7, 12-dimethylbenzanthracene
CYLD	cylindromatosis
DUB	deubiquitinase
HA	hemagglutinin A
H&E	hematoxylin & eosin
HF	hair follicles
K5	keratin 5
NMSC	non melanoma skin cancer
ORS	outer root sheath
SRF	Serum Response Factor
SCC	squamous cell carcinomas
Sma	α-smooth muscle actin
SEM	standard error of the mean
TNF-α	tumor necrosis factor-α
TPA	12-O-tetradecanoylphorbol-13-acetate
WB	Western blot

## References

[1] Mathis J.B., Lai Y., Qu C., Janicki S.J., Cui T. (2015). CYLD-mediated signaling and diseases. Curr. Drug Targets.

[2] Brummelkamp T.R., Nijman S.M., Dirac A.M., Bernards R. (2003). Loss of the cylindromatosis tumour suppressor inhibits apoptosis by activating NF-kappaB. Nature.

[3] Kovalenko A., Chable-Bessia C., Cantarella G., Israel A., Wallach D., Courtois G. (2003). The tumour suppressor CYLD negatively regulates NF-kappaB signalling by deubiquitination. Nature.

[4] Trompouki E., Hatzivassiliou E., Tsichritzis T., Farmer H., Ashworth A., Mosialos G. (2003). CYLD is a deubiquitinating enzyme that negatively regulates NF-kappaB activation by TNFR family members. Nature.

[5] AACR Project Genie Consortium (2017). AACR Project GENIE: Powering Precision Medicine through an International Consortium. Cancer Discov..

[6] Hellerbrand C., Bumes E., Bataille F., Dietmaier W., Massoumi R., Bosserhoff A.K. (2007). Reduced expression of CYLD in human colon and hepatocellular carcinomas. Carcinogenesis.

[7] Kinoshita H., Okabe H., Beppu T., Chikamoto A., Hayashi H., Imai K., Mima K., Nakagawa S., Yokoyama N., Ishiko T. (2013). CYLD downregulation is correlated with tumor development in patients with hepatocellular carcinoma Down-regulation of the Tumor Suppressor CYLD Enhances the Transformed Phenotype of Human Breast Cancer Cells. Mol. Clin. Oncol..

[8] Orfanidou T., Xanthopoulos K., Dafou D., Pseftogas A., Hadweh P., Psyllaki C., Hatzivassiliou E., Mosialos G. (2017). Down-regulation of the Tumor Suppressor CYLD Enhances the Transformed Phenotype of Human Breast Cancer Cells. Anticancer Res..

[9] Fuchs E. (1990). Epidermal differentiation: The bare essentials. J. Cell Biol..

[10] Alameda J.P., Moreno-Maldonado R., Navarro M., Bravo A., Ramirez A., Page A., Jorcano J.L., Fernandez-Acenero M.J., Casanova M.L. (2010). An inactivating CYLD mutation promotes skin tumor progression by conferring enhanced proliferative, survival and angiogenic properties to epidermal cancer cells. Oncogene.

[11] Alameda J.P., Ramirez A., Garcia-Fernandez R.A., Navarro M., Page A., Segovia J.C., Sanchez R., Suarez-Cabrera C., Paramio J.M., Bravo A. (2019). Premature aging and cancer development in transgenic mice lacking functional CYLD. Aging.

[12] de Marval P.M., Lutfeali S., Jin J.Y., Leshin B., Selim M.A., Zhang J.Y. (2011). CYLD Inhibits Tumorigenesis and Metastasis by Blocking JNK/AP1 Signaling at Multiple Levels. Cancer Prev. Res..

[13] Masoumi K.C., Shaw-Hallgren G., Massoumi R. (2011). Tumor Suppressor Function of CYLD in Nonmelanoma Skin Cancer. J. Skin Cancer.

[14] Limmer B.L. (2001). Nonmelanoma skin cancer: Today’s epidemic. Tex. Med..

[15] Karia P.S., Han J., Schmults C.D. (2013). Cutaneous squamous cell carcinoma: Estimated incidence of disease, nodal metastasis, and deaths from disease in the United States, 2012. J. Am. Acad. Dermatol..

[16] Christenson L.J., Borrowman T.A., Vachon C.M., Tollefson M.M., Otley C.C., Weaver A.L., Roenigk R.K. (2005). Incidence of basal cell and squamous cell carcinomas in a population younger than 40 years. JAMA.

[17] Ghosh S., Karin M. (2002). Missing pieces in the NF-kappaB puzzle. Cell.

[18] Mukhopadhyay T., Roth J.A., Maxwell S.A. (1995). Altered expression of the p50 subunit of the NF-kappa B transcription factor complex in non-small cell lung carcinoma. Oncogene.

[19] Nair A., Venkatraman M., Maliekal T.T., Nair B., Karunagaran D. (2003). NF-kappaB is constitutively activated in high-grade squamous intraepithelial lesions and squamous cell carcinomas of the human uterine cervix. Oncogene.

[20] Neri A., Chang C.C., Lombardi L., Salina M., Corradini P., Maiolo A.T., Chaganti R.S., Dalla-Favera R. (1991). B cell lymphoma-associated chromosomal translocation involves candidate oncogene lyt-10, homologous to NF-kappa B p50. Cell.

[21] Sau A., Lau R., Cabrita M.A., Nolan E., Crooks P.A., Visvader J.E., Pratt M.A. (2016). Persistent Activation of NF-κB in BRCA1-Deficient Mammary Progenitors Drives Aberrant Proliferation and Accumulation of DNA Damage. Cell Stem Cell.

[22] Thornburg N.J., Pathmanathan R., Raab-Traub N. (2003). Activation of nuclear factor-kappaB p50 homodimer/Bcl-3 complexes in nasopharyngeal carcinoma. Cancer Res..

[23] Walther W., Kobelt D., Bauer L., Aumann J., Stein U. (2015). Chemosensitization by diverging modulation by short-term and long-term TNF-α action on ABCB1 expression and NF-κB signaling in colon cancer. Int. J. Oncol..

[24] Baud V., Karin M. (2009). Is NF-kappaB a good target for cancer therapy? Hopes and pitfalls. Nat. Rev. Drug Discov..

[25] Budunova I.V., Perez P., Vaden V.R., Spiegelman V.S., Slaga T.J., Jorcano J.L. (1999). Increased expression of p50-NF-kappaB and constitutive activation of NF-kappaB transcription factors during mouse skin carcinogenesis. Oncogene.

[26] Kim C., Pasparakis M. (2014). Epidermal p65/NF-kappaB signalling is essential for skin carcinogenesis. EMBO Mol. Med..

[27] Kobielak A., Fuchs E. (2006). Links between alpha-catenin, NF-kappaB, and squamous cell carcinoma in skin. Proc. Natl. Acad. Sci. USA.

[28] Larcher F., Murillas R., Bolontrade M., Conti C.J., Jorcano J.L. (1998). VEGF/VPF overexpression in skin of transgenic mice induces angiogenesis, vascular hyperpermeability and accelerated tumor development. Oncogene.

[29] Mueller M.M. (2006). Inflammation in epithelial skin tumours: Old stories and new ideas. Eur. J. Cancer.

[30] Alameda J.P., Fernández-Aceñero M.J., Moreno-Maldonado R., Navarro M., Quintana R., Page A., Ramírez A., Bravo A., Casanova M.L. (2011). CYLD regulates keratinocyte differentiation and skin cancer progression in humans. Cell Death Dis..

[31] Zhang L.M., Zhou J.J., Luo C.L. (2018). CYLD suppression enhances the pro-inflammatory effects and hyperproliferation of rheumatoid arthritis fibroblast-like synoviocytes by enhancing NF-κB activation. Arthritis Res. Ther..

[32] Massoumi R., Chmielarska K., Hennecke K., Pfeifer A., Fässler R. (2006). Cyld inhibits tumor cell proliferation by blocking Bcl-3-dependent NF-kappaB signaling. Cell.

[33] Ramírez A., Bravo A., Jorcano J.L., Vidal M. (1994). Sequences 5’ of the bovine keratin 5 gene direct tissue- and cell-type-specific expression of a lacZ gene in the adult and during development. Differentiation.

[34] Page A., Navarro M., Garin M., Perez P., Casanova M.L., Moreno R., Jorcano J.L., Cascallana J.L., Bravo A., Ramirez A. (2010). IKKbeta leads to an inflammatory skin disease resembling interface dermatitis. J. Investig. Dermatol..

[35] Alameda J.P., Gaspar M., Ramirez A., Navarro M., Page A., Suarez-Cabrera C., Fernandez M.G., Merida J.R., Paramio J.M., Garcia-Fernandez R.A. (2016). Deciphering the role of nuclear and cytoplasmic IKKalpha in skin cancer. Oncotarget.

[36] Leder A., Kuo A., Cardiff R.D., Sinn E., Leder P. (1990). v-Ha-ras transgene abrogates the initiation step in mouse skin tumorigenesis: Effects of phorbol esters and retinoic acid. Proc. Natl. Acad. Sci. USA.

[37] Filler R.B., Roberts S.J., Girardi M. (2007). Cutaneous two-stage chemical carcinogenesis. CSH Protoc..

[38] Alameda J.P., Moreno-Maldonado R., Fernandez-Acenero M.J., Navarro M., Page A., Jorcano J.L., Bravo A., Ramirez A., Casanova M.L. (2011). Increased IKKalpha Expression in the Basal Layer of the Epidermis of Transgenic Mice Enhances the Malignant Potential of Skin Tumors. PLoS ONE.

[39] Toll A., Margalef P., Masferrer E., Ferrandiz-Pulido C., Gimeno J., Pujol R.M., Bigas A., Espinosa L. (2015). Active nuclear IKK correlates with metastatic risk in cutaneous squamous cell carcinoma. Arch. Dermatol. Res..

[40] Alameda J.P., Fernandez-Acenero M.J., Quintana R.M., Page A., Ramirez A., Navarro M., Casanova M.L. (2013). Functional inactivation of CYLD promotes the metastatic potential of tumor epidermal cells. J. Investig. Dermatol..

[41] Kauvar A.N., Arpey C.J., Hruza G., Olbricht S.M., Bennett R., Mahmoud B.H. (2015). Consensus for Nonmelanoma Skin Cancer Treatment, Part II: Squamous Cell Carcinoma, Including a Cost Analysis of Treatment Methods. Dermatol. Surg..

[42] Bolontrade M.F., Stern M.C., Binder R.L., Zenklusen J.C., Gimenez-Conti I.B., Conti C.J. (1998). Angiogenesis is an early event in the development of chemically induced skin tumors. Carcinogenesis.

[43] Chaturvedi M.M., Sung B., Yadav V.R., Kannappan R., Aggarwal B.B. (2011). NF-κB addiction and its role in cancer: ‘one size does not fit all’. Oncogene.

[44] Hoesel B., Schmid J.A. (2013). The complexity of NF-κB signaling in inflammation and cancer. Mol. Cancer.

[45] Mantovani A., Allavena P., Sica A., Balkwill F. (2008). Cancer-related inflammation. Nature.

[46] Casanova M.L., Larcher F., Casanova B., Murillas R., Fernandez-Acenero M.J., Villanueva C., Martinez-Palacio J., Ullrich A., Conti C.J., Jorcano J.L. (2002). A critical role for ras-mediated, epidermal growth factor receptor-dependent angiogenesis in mouse skin carcinogenesis. Cancer Res..

[47] O’Donnell M.A., Perez-Jimenez E., Oberst A., Ng A., Massoumi R., Xavier R., Green D.R., Ting A.T. (2011). Caspase 8 inhibits programmed necrosis by processing CYLD. Nat. Cell Biol..

[48] Müller I., Strozyk E., Schindler S., Beissert S., Oo H.Z., Sauter T., Lucarelli P., Raeth S., Hausser A., Al Nakouzi N. (2020). Cancer Cells Employ Nuclear Caspase-8 to Overcome the p53-Dependent G2/M Checkpoint through Cleavage of USP28. Mol. Cell.

[49] Liang G., Ahlqvist K., Pannem R., Posern G., Massoumi R. (2011). Serum response factor controls CYLD expression via MAPK signaling pathway. PLoS ONE.

[50] Kiss A., Koppel A.C., Anders J., Cataisson C., Yuspa S.H., Blumenberg M., Efimova T. (2016). Keratinocyte p38δ loss inhibits Ras-induced tumor formation, while systemic p38δ loss enhances skin inflammation in the early phase of chemical carcinogenesis in mouse skin. Mol. Carcinog..

[51] Kiss A., Koppel A.C., Murphy E., Sall M., Barlas M., Kissling G., Efimova T. (2019). Cell Type-Specific p38δ Targeting Reveals a Context-, Stage-, and Sex-Dependent Regulation of Skin Carcinogenesis. Int. J. Mol. Sci..

[52] Schindler E.M., Hindes A., Gribben E.L., Burns C.J., Yin Y., Lin M.H., Owen R.J., Longmore G.D., Kissling G.E., Arthur J.S. (2009). p38delta Mitogen-activated protein kinase is essential for skin tumor development in mice. Cancer Res..

[53] Gupta S.C., Kim J.H., Prasad S., Aggarwal B.B. (2010). Regulation of survival, proliferation, invasion, angiogenesis, and metastasis of tumor cells through modulation of inflammatory pathways by nutraceuticals. Cancer Metastasis Rev..

[54] Vogelstein B., Kinzler K.W. (2004). Cancer genes and the pathways they control. Nat. Med..

