# Supplementary figures and images for: CYLD Inhibits the Development of Skin Squamous Cell Tumors in Immunocompetent Mice
Source: Int J Mol Sci. 2021 Jun 23;22(13):6736. doi: 10.3390/ijms22136736 (PMC8268443; doi:10.3390/ijms22136736)

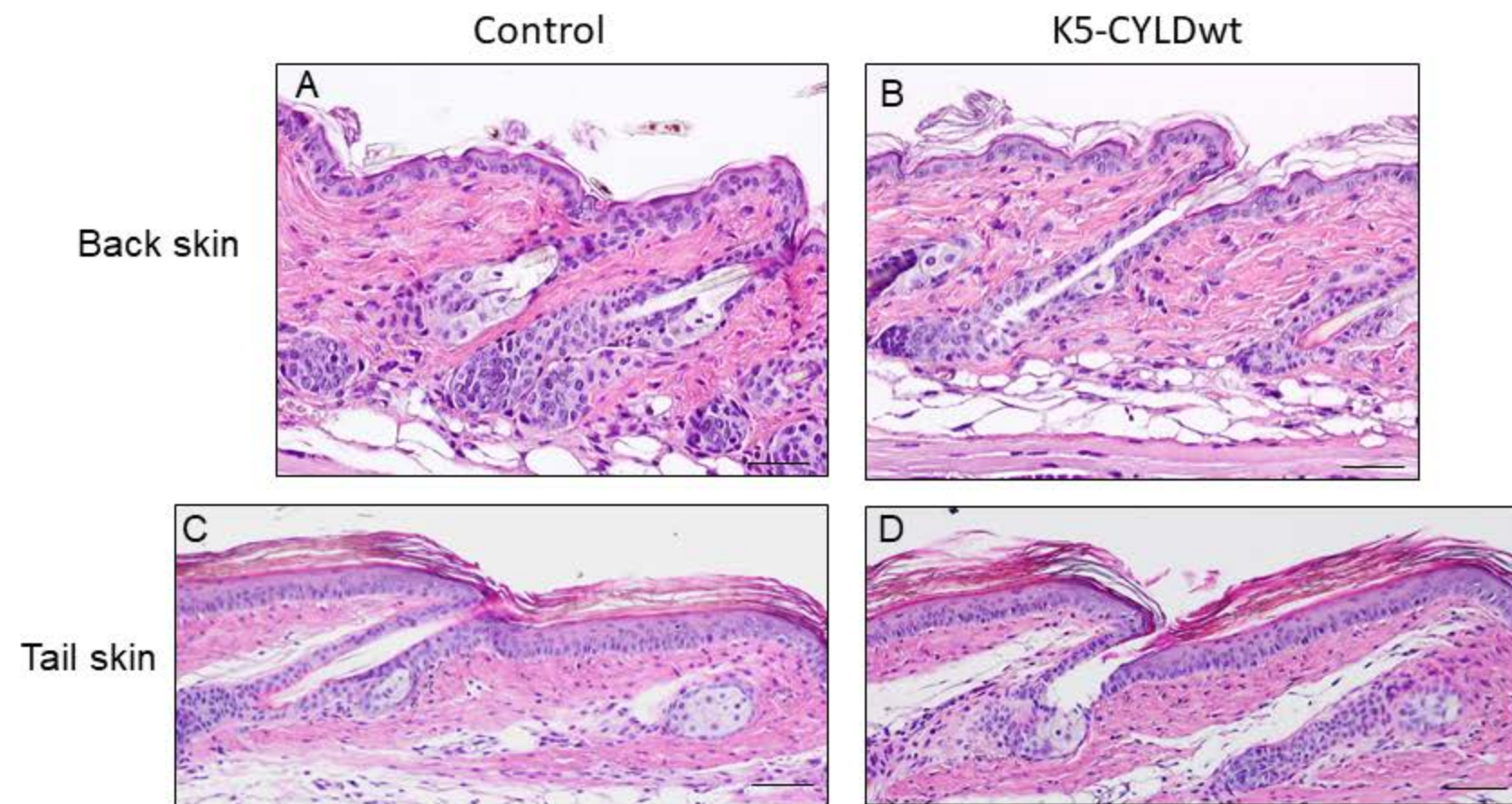

Figure S1

Supplement: Supplementary file 1 [file ijms-22-06736-s001.zip › Figure S1.pdf]
